# Using segmented regression analysis of interrupted time series data to assess colonoscopy quality outcomes of a web-enhanced implementation toolkit to support evidence-based practices for bowel preparation: a study protocol

**DOI:** 10.1186/s13012-015-0276-3

**Published:** 2015-06-07

**Authors:** Alex T. Ramsey, Julia Maki, Beth Prusaczyk, Yan Yan, Jean Wang, Rebecca Lobb

**Affiliations:** 1 Washington University Brown School of Social Work, 1 Brookings Dr., St. Louis, MO 63130 USA; 2 Washington University School of Medicine, 660 S. Euclid Ave, St. Louis, MO 63110 USA

**Keywords:** Colonoscopy care, Evidence-based practice, Implementation strategies, Web-based, Toolkits, Pragmatic trial, PRECIS

## Abstract

**Background:**

While there is convincing evidence on interventions to improve bowel preparation for patients, the evidence on how to implement these evidence-based practices (EBPs) in outpatient colonoscopy settings is less certain. The Strategies to Improve Colonoscopy (STIC) study compares the effect of two implementation strategies, physician education alone versus physician education plus an implementation toolkit for staff, on adoption of three EBPs (split-dosing of bowel preparation, low-literacy education, teach-back) to improve pre-procedure and intra-procedure quality measures. The implementation toolkit contains a staff education module, website containing tools to support staff in delivering EBPs, tailored patient education materials, and brief consultation with staff to determine how the EBPs can be integrated into the existing workflow. Given adaptations to the implementation plan and intentional flexibility in the delivery of the EBPs, we utilize a pragmatic study to balance external validity with demonstrating effectiveness of the implementation strategies.

**Methods/Design:**

Participants will include all outpatient colonoscopy physicians, staff, and patients from a convenience sample of six endoscopy settings. Aim #1 will explore the relative effect of two strategies to implement patient-level EBPs on adoption and clinical quality outcomes. We will assess the change in level and trends of clinical quality outcomes (i.e., adequacy of bowel preparation, adenoma detection) using segmented regression analysis of interrupted time series data with two groups (intervention and delayed start). Aim #2 will examine the influence of organizational readiness to change on EBP implementation. We use a PRECIS diagram to reflect the extent to which each indicator of the study was pragmatic versus explanatory, revealing a largely pragmatic study.

**Discussion:**

Implementation challenges have already motivated several adaptations to the original plan, reflecting the nature of implementation in real-world healthcare settings. The pragmatic study responds to the evolving needs of its healthcare partners and allows for flexibility in intervention delivery, thereby informing clinical decision-making in real-world settings. The current study will provide information about *what* works (intervention effectiveness), *for whom* it works (influence of Medicaid versus other insurance), in *which contexts* it works (setting characteristics that influence implementation), and *how* it works best (comparison of implementation strategies).

**Electronic supplementary material:**

The online version of this article (doi:10.1186/s13012-015-0276-3) contains supplementary material, which is available to authorized users.

## Background

In 2002, more than 14 million colonoscopies were performed in the United States of America [1] and annual procedures are on the rise [2] due to increased physician recommendation [3, 4] and patient preferences for colonoscopy, over fecal occult blood test or sigmoidoscopy for colorectal cancer screening [2]. Inadequate bowel preparation and substantial variability in adenoma detection [5–7] from 7.5 to 33.3 % [6, 8] are critical colonoscopy quality issues [5]. To improve the quality of colonoscopy, the American Society for Gastrointestinal Endoscopy and the American College of Gastroenterology recommend that for asymptomatic patients more than 50 years of age, adenoma detection should be >30 % for men and >20 % for women [5, 9], and inadequate bowel preparation should be <15 % [10, 11]. Inadequate bowel preparation is a primary cause of low adenoma detection [12] and can occur in over 30 % of patients, or 4.2 million persons per year [13]. In addition, inadequate bowel preparation increases complication rates [14] and cost of colonoscopy by 12–22 % [12]. Patients’ age, sex, and comorbidities are associated with adequacy of bowel preparation [6]. However, Medicaid insurance is among the strongest risk factors for inadequate bowel preparation (odds ratios 1.846–8.707 compared to other insurance). Given that only 39 % of Medicaid-insured adults read at or above an 8th grade level, low-literacy education for bowel preparation might be particularly beneficial to the quality of colonoscopy for patients insured by Medicaid [15].

Despite these grim statistics, randomized controlled trials of two interventions (one clinical and one behavioral) hold promise for improving bowel preparation and adenoma detection. Irrespective of the bowel preparation [16], *split-dosing* can improve the adequacy of bowel preparation by 10 percentage points (*p* < 0.0001) and adenoma detection by six percentage points (*p* < 0.001) and is more highly accepted [17] by patients compared to taking a full dose on the same day [18–20]. In addition, an almost fourfold improvement in adequacy of bowel preparation has been found among patients who receive *low-literacy education* compared to education written for 6th grade or higher [21]. However, written instructions are more effective when combined with verbal instructions [18, 22], signaling the importance of confirming patient understanding of bowel preparation instructions. Representing a third evidence-based practice (EBP) for improving bowel preparation, *teach-back* is the practice of reviewing instructions with patients, ensuring that patients can repeat key points (i.e., teach back), and addressing any barriers to completion of instructions. Both low-literacy education and teach-back are in accordance with the Agency for Healthcare Research and Quality’s and the National Quality Council’s recommendation for using universal precautions to improve health literacy in all patients.

While there is convincing evidence on interventions to improve bowel preparation for patients, the evidence on how to implement these patient interventions in outpatient colonoscopy settings is less obvious. Outpatient colonoscopy requires relationship-centered care [23] where staff provides patients with education for bowel preparation that reflects the physician’s choice of bowel preparation medication and dose to maximize the potential for a clean colon on the day of the procedure. The structure and qualities of the physician-staff relationship for outpatient colonoscopy remain unstudied. Yet understanding the qualities and patterns of these relationships can inform the implementation of changes to patient procedures to improve bowel preparation prior to outpatient colonoscopy [23]. Our study fills this research gap in a pragmatic study that examines the effect of implementation strategies directed toward physicians and staff in endoscopy settings on implementation of EBPs to improve bowel preparation for patients overall and for those insured by Medicaid. We also leverage the pragmatic study to conduct formative research about theory-informed aspects of endoscopy settings (e.g., readiness to change) that influence implementation of patient interventions. These healthcare quality and implementation issues are of critical importance to decision-makers in health service organizations because practice-based stakeholders are under increased pressure by Medicare and Medicaid to improve quality of care and reduce cost.

**Fig. 1:**
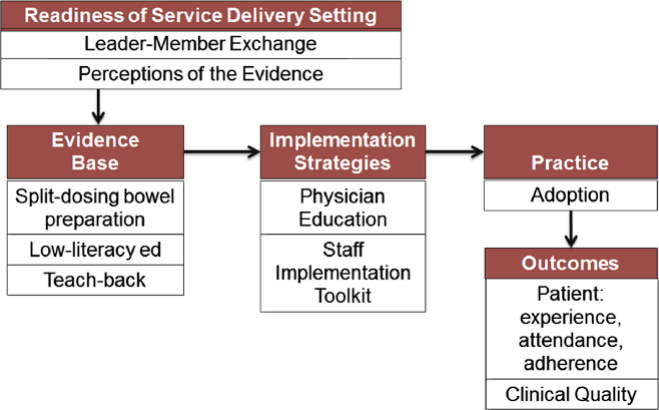
Conceptual model of STIC study. Adapted from the Proctor et al. conceptual model of implementation research [36, 37]

Addressing barriers to implementation of EBPs for bowel preparation can help reduce the substantial variation in colonoscopy outcomes found across healthcare settings. Several frameworks of implementation for interventions to improve health have emerged over the last decade [24, 25]. The Promoting Action on Research Implementation in Health Services (PARIHS) framework is among the most researched frameworks of implementation [26]. There is evidence to suggest that key factors of the PARIHS framework—perceptions of the evidence, context, and facilitation—are critical precursors to successful implementation of complex changes in healthcare settings (Fig. 1) [27, 24]. Two aspects of scientific evidence, relative advantage of the new intervention over an existing intervention [27] and compatibility with existing values and past experiences, are strongly correlated with adoption of interventions [28, 29]. Context refers to organizational culture and leadership [29, 25]. These contextual factors affect staffs’ capacity to facilitate implementation by encouraging analytical engagement in quality improvement processes [30]. Facilitation refers to the structures and processes in place to support change in clinical practice [29, 31]. When implementation efforts are interdependent across multiple levels of healthcare providers, as is expected in this study, context and facilitation are highly influenced by the structure and quality of the physician-staff relationship. As informed by leader-member exchange (LMX) theory, employees (i.e., staff) and supervisors (i.e., physicians) establish unique exchange relationships that contribute to employee work attitudes and performance (i.e., implementation of evidence-based colonoscopy care) [32]. Together, evidence, context, and facilitation contribute to organizations’ readiness to successfully implement change [24, 31]. Published reports on complex change initiatives suggest a median implementation success rate of only 33 % [24] due to factors such as false start of an intervention, staff resistance to implementation, or failure to implement an intervention [27]. Understanding the structure and quality of relationships between the physicians and implementers of patient education in endoscopy settings is important to developing strategies to increase adoption of EBPs. Our study 1) compares the effect of two implementation strategies on the adoption of EBPs to improve patient bowel preparation and 2) examines whether physician-team level aspects of readiness to change are associated with colonoscopy quality, patient, and implementation outcomes to gain a better understanding of the nature of implementation of pre-colonoscopy patient interventions in endoscopy settings.

### Present study

The Strategies to Improve Colonoscopy (STIC) study compares the effect of two implementation strategies, physician education alone versus physician education *plus* an implementation toolkit for staff (i.e., education, website, tailored patient education materials, and a brief consultation), on adoption of three EBPs to improve pre-procedure quality measures (patient experience, adequacy of bowel preparation) and intra-procedure quality measures (cecal intubation, adenoma detection). The EBPs are as follows: 1) split-dosing of bowel preparation, 2) low-literacy education, and 3) teach-back. The goal of the physician education is to improve perceptions of the EBPs and to motivate change among physicians [33], whereas the goal of the implementation toolkit is to improve perceptions of the EBPs, motivate change, and *enable change* among staff [30]. The toolkit will assist endoscopy staff to implement the three EBPs to improve education for bowel preparation for patients. The implementation toolkit contains 1) staff education module, 2) a website containing tools to support staff in learning teach-back and providing low-literacy education materials for patients, 3) patient education materials including a 5-month supply of printed patient education brochures tailored to their site and bowel preparation preferences, and a laminated pocket card and poster to remind and help staff to use teach-back, and 4) a brief consultation with study staff to determine how the EBPs can be integrated into the existing workflow. Education alone is a weak strategy to change physician behavior [34, 35], and there is limited evidence on the effect of implementation toolkits on staff implementation of EBPs. Therefore, the STIC study aims to test the effect of physician education versus physician education plus an implementation toolkit for staff on the implementation and quality outcomes. In light of several adaptations made to the implementation plan and the intentional flexibility in the delivery of the EBPs, this study utilizes a pragmatic study to balance external validity with demonstrated effectiveness of the implementation strategies.

## Methods/Design

### Strategic partners

Our strategic partners include physician champions from six endoscopy settings in the St. Louis area. All settings are affiliated with BJC HealthCare, one of the largest non-profit healthcare organizations in the United States of America with 13 hospitals. Despite the affiliation, each hospital is run as a separate organization. Quality improvement initiatives are not routinely implemented across BJC. However, BJC has a Center for Clinical Excellence that provides decision support, analytic teams, and performance improvement services to affiliated hospitals, and they have a strong interest in learning how to implement widespread change throughout BJC to improve quality of care. Two of the settings are teaching hospitals where colonoscopies are performed by physicians from the university. At two other partnering hospitals, colonoscopies are performed by physicians in private practice.

### Study overview

We adapted Proctor et al.’s conceptual model of implementation evaluation to show the relationships among factors that influence variability in success of quality improvement of colonoscopy (Fig. 1) [36, 37]. Aim #1 will explore the relative effect of two strategies to implement patient-level EBPs on quality outcomes. Aim #2 will assess aspects of endoscopy settings (i.e., perceptions of the evidence, the quality and structure of the relationship between physician and implementers) that influence implementation, patient, and colonoscopy quality outcomes in endoscopy settings.

#### Aim #1

Compare the effect of physician education versus physician education plus a staff implementation toolkit on staff adoption of evidence-based practices and colonoscopy quality outcomes (i.e., adequacy of bowel preparation, adenoma detection).

To accomplish aim #1, we will compare change in colonoscopy quality outcomes from before to after implementation of interventions for the two study groups: a) overall and b) Medicaid versus other insurance using administrative and medical record data with interrupted time series analysis.

### Settings and subjects

**Fig. 2:**
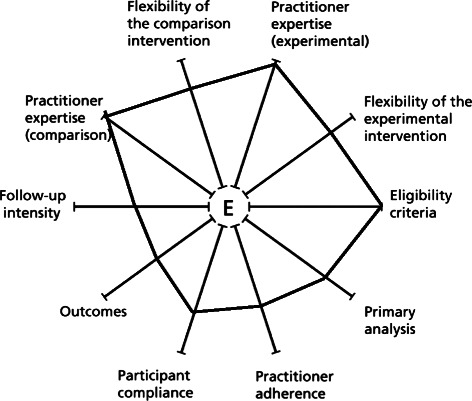
PRECIS diagram of the pragmatic-explanatory continuum for the STIC study. The study becomes more pragmatic (and less explanatory) as each indicator point moves away from the central “E”

This study will be conducted with a convenience sample of six endoscopy settings. Our study is highly pragmatic with regard to eligibility. We will invite all 70 physicians that perform outpatient colonoscopy, regardless of their specialty (i.e., gastroenterology, surgery), and all of their staff that provide bowel preparation education to patients before outpatient colonoscopy. All patients scheduled for outpatient colonoscopy will be included, regardless of whether they are scheduled for a screening or diagnostic procedure. The PRECIS diagram illustrated in Fig. 2 reflects the extent to which each indicator of the study was pragmatic versus explanatory. Table 1 explains the PRECIS diagram by providing an assessment of each PRECIS domain for this study [38]. Our strategic partners will facilitate recruitment of physicians by co-signing recruitment letters and, if recruitment challenges occurs, by sending emails, calling physicians, and introducing the study at department meetings. Based on previous experience with physician recruitment to studies, we anticipate participation from at least 80 % of eligible physicians (70*0.80 = 56 physicians) and that each physician will have at least two staff that provide education for bowel preparation (56*2 = *n* = 112 staff). To minimize the potential for diffusion of treatment to our comparison group, we will assign physicians’ staff either to usual care or to receive the implementation toolkit. Physicians in both study groups will receive education. Assignment will be based on baseline adequacy of bowel preparation, balance of patient units and structure of physician-staff relationship. Over 10,000 outpatient colonoscopies per year are performed in our sample. Overall, 23 % of patients are covered by Medicaid. We anticipate inclusion of more than 10,000 patients in the study (20,000 over 2 years*15/24 months observation = 12,500*0.80 minimum physician participation = 10,000 total, >600 per month).

**Table 1:** PRECIS assessment of the STIC study

PRECIS domains	Assessment of domain
Participant eligibility criteria	Eligible participants will include all physicians performing outpatient colonoscopy regardless of specialty, all staff providing bowel preparation education to patients before outpatient colonoscopy, and all patients scheduled for outpatient colonoscopy regardless of whether they are scheduled for a screening or diagnostic procedure.
Experimental intervention—flexibility	The experimental condition will allow for flexibility in the timing of staff delivering the intervention to patients so that the EBPs are integrated into existing processes as seamlessly as possible. However, staff may be provided with recommendations for the timing of intervention delivery to patients.
Experimental intervention—practitioner expertise	All physicians and staff members will be involved regardless of specialization, level of training, or expertise.
Comparison intervention(s)—flexibility	Physicians will receive an education module, but physicians’ staff members will be assigned to usual practice.
Comparison intervention(s)—practitioner expertise	All physicians and staff members will be involved regardless of specialization, level of training, or expertise.
Follow-up intensity	To boost the response rate above, initial mailings will include contacts from our strategic partners, and study staff will follow up weekly with email reminders and phone calls to non-respondents. After enrollment, no contact is made with physicians or staff.
Primary trial outcome	Primary outcomes will include both an intermediate implementation outcome (i.e., EBP adoption) and more long-range clinical outcomes (i.e., bowel preparation, adenoma detection). The outcomes, particularly the clinical outcomes, are well-specified, clinically meaningful, and assessed under usual conditions.
Participant compliance with intervention	Physician participation in the education module and provision of staff names, as well as staff participation rates for each component of the toolkit (i.e., education module, website, ordering materials, brief consultation) will be measured through Google Analytics and Qualtrics primarily for descriptive purposes and to inform improvement of the toolkit in future studies. No other compliance-improving strategies will be applied in this study.
Practitioner adherence to study protocol	The provision of teach-back and low-literacy education to patients will be measured and assessed but not fed back to staff or physicians during the course of the study. No ongoing data feedback or other adherence-improving strategies will be applied in this study.
Analysis of primary outcome	The segmented regression analysis of interrupted time series data (SRAITSD) will maximize external validity by informing the generalizability of effects across endoscopy settings and insurance strata.

### Delivery of patient interventions

We will allow for flexibility in the timing of staff delivering the intervention to patients so that the EBPs are integrated into existing processes as seamless as possible. However, when relevant during consultations (e.g., staff seeking advice), we will provide staff with recommendations for the timing of intervention delivery to patients. We will recommend that clinical staff mail the instructions for bowel preparation with an appointment reminder approximately 2 weeks prior to the procedure and call patients to use the teach-back method 1 week prior to the procedure.

### Implementation strategies

The primary aim of this study is to test the additive effect of physician education plus an implementation toolkit (compared to physician education alone) for endoscopy staff providing pre-colonoscopy bowel preparation education to patients on colonoscopy quality outcomes (i.e., adequacy of bowel preparation, adenoma detection). The implementation toolkit consists of four components: 1) *staff education module* including clinical goals for colonoscopy quality for adequacy of bowel preparation, adenoma detection, and cecal intubation, 2) *staff support website*, including information on the EBPs of split-dosing, teach-back, and low-literacy education, 3) *patient education materials*, including a 5-month supply of printed patient education brochures tailored to their practice and bowel preparation of their choice, plus a laminated pocket card and poster to remind and support staff to use teach-back, and 4) *brief consultation* with study staff to identify how EBPs can be integrated into existing workflow. The comparison group will receive the physician education module, but not the staff implementation toolkit.

### Measures

The Center for Clinical Excellence at BJC will obtain all data required for aim #1 from clinical and administrative systems at the endoscopy settings. The primary outcomes for aim #1 are adoption of EBPs, adequacy of bowel preparation, and adenoma detection. We will measure adoption of the EBPs (i.e., split-dosing, use of low-literacy education materials, teach-back) using a structured survey administered to staff and patients at baseline and follow-up. Sample questions on adoption will assess whether staff asked patients to “take half of the bowel preparation medication” (the day before/the morning of the test), with response options including “No,” “Yes,” and “I don’t know/Not applicable.” Questions will also be asked about whether or not staff provided the recommended low-literacy paper instructions and whether or not staff asked patients to “repeat instructions for bowel preparation in their own words.” Both clinical outcomes (adequacy of bowel preparation, adenoma detection) are standard elements of colonoscopy reports [10], and earlier research suggests that these data are routinely recorded in electronic records [39]. Bowel preparation is commonly described using the Aronchick scale (Kappa ICC 0.77) [40] as excellent, good, fair, or poor. Excellent or good ratings are considered adequate [5, 39]. Excellent refers to no or minimal solid stool and only small amounts of clear fluid requiring suctioning. Good refers to no or minimal solid stool with large amounts of clear fluid requiring suctioning. Fair refers to collections of semisolid debris that are cleared with difficulty. Poor refers to solid or semisolid debris that cannot be effectively cleared. We will also capture information about factors that are associated with adequacy of bowel preparation and adenoma detection including characteristics of the patient (age, gender, type of insurance, co-morbid conditions (i.e., history of constipation, diabetes), body mass index, use of narcotic medications) [41], primary language [14], and procedure (i.e., physician specialty, type of bowel preparation, indication for procedure, date of procedure [8]).

### Analysis

To assess change in clinical outcomes, we will use segmented regression analysis of interrupted time series data (SRAITSD). SRAITSD is an excellent analysis method for pragmatic studies because it minimizes threats to internal validity while maximizing external validity. For our application, individual patient outcomes are aggregated into proportion of adequate bowel preparation or proportion with adenoma detection in 2-week intervals, the time representing our unit of analysis. A sequence of the proportions over time generates time series data, with segments separated by two different interventions (i.e., comparative effectiveness and a replication study). The observation period for the time series analysis (Table 2) consists of three segments: baseline, comparative-effectiveness intervention, and replication study. Each segment is ~5 months in length. Two parameters define each segment of a time series: level and trend (see Additional file 1 for additional information on the analytic model) [42]. This model can be fit and tested using SAS software. We have a sufficient number of observations per segment (10 observations) and individual patient outcomes per observation (>100 units) for robust estimates of change in level and trend for outcomes [42, 43].

**Table 2:** Interrupted time series study design—15-month observation period

Segments	Baseline			Comparative-effectiveness				Replication			
Group 1	O_1_	…	O_10_	X_1_	O_11_	…	O_20_	X_1_X_2_	O_21_	…	O_30_
Group 2	O_1_	…	O_10_	X_1_X_2_	O_11_	…	…	…	…	…	O_30_

*Note*: Each observation represents a 2-week interval. X1 = physician education, *X*2 = physician education plus staff training and support

To test intervention effects in each group, we first examine the plots of 2-week proportions over time. Then, we will fit the model separately in each group. β2 and β3 are the change in level and in trend of outcome, respectively, due to the comparative-effectiveness intervention. β4 and β5, which only apply to group 1, are the change in level and in trend of outcome, respectively, due to the replication study beyond the comparative-effectiveness intervention. For each group, statistical significance of these changes can be tested using the ratios of these regression coefficients to their estimated standard errors (*p* value). Because our study adopts a parallel design, we can also examine the statistical significance of the difference in these changes across groups by computing the difference in the regression coefficients divided by a standard error of the coefficient difference (*p* value), which can be easily obtained from standard errors of the regression coefficients in two separate models. A statistically significant (*p* < 0.05) difference in change in the level of outcome for group 1 versus group 2 will inform us about the comparative effectiveness of the physician education versus the physician education *plus* implementation toolkit. Alternatively, a statistically significant (*p* < 0.05) difference in trends of outcomes for group 1 versus group 2 will inform us about potential threats to validity due to outside events or program drift that occurs when fidelity to implementation protocols weaken over time. Finally, a statistically significant (*p* < 0.05) difference in the level of outcomes for group 1 in segment 2 versus group 1 in segment 3 will inform us about the generalizability of the comparative-effectiveness study effects across endoscopy settings. In order to examine modifying intervention effect by insurance status, we will stratify our study subjects by Medicaid versus other insurance status. Therefore, we will have 4 time series—two groups by two insurance strata. These series have the same time definition and the same segments, so the regression coefficients have the same interpretation. We will fit SRAITSD to each of the 4 time series and compare the relevant regression coefficients between Medicaid versus other insurance.

To assess the extent to which the implementation toolkit influenced adoption of EBPs over time, we will model adoption at follow-up as a function of study group assignment, controlling for the baseline measure of adoption. We will assess adoption for each EBP (i.e., split-dosing, low-literacy education, teach-back) separately and use separate statistical models for adoption measured from the perspective of the following: 1) staff, 2) physicians, and 3) patients. The regression coefficient for study group assignment will quantify the effect of different approaches on the outcome.

#### Aim #2

Examine aspects of organizational readiness to change that facilitate or deter implementation of patient interventions to improve quality of colonoscopy

To accomplish aim #2, we will identify aspects of readiness to change (i.e., evidence, context, facilitation) associated with implementation (i.e., fidelity, dose, cost), patient (i.e., experience, attendance, adherence), and colonoscopy quality outcomes (aim #1) using structured surveys.

### Subjects

We will recruit physician teams and patient-level subjects for aim #2. The physician team will include physicians recruited for aim #1 and their staff who provide pre-colonoscopy education to patients. We anticipate recruiting a total of 56 physician teams (70 physicians total*0.80 participation) with at least two staff respondents per physician team (56*2 = 112 staff). The study coordinator will send an email to physician team members including a link to an online survey and invitation to participate. The survey invitation will include a personal online code that physician team members can use to complete the online survey. To boost the response rate above, initial mailings will include contacts from our strategic partners, and study staff will follow up weekly with email reminders and phone calls to non-respondents.

### Measures

We will measure both implementation and patient outcomes for aim #2. Implementation outcomes include fidelity, dose, and cost. Fidelity will be measured as the percent of intervention activities delivered as planned with regard to receipt of the tailored patient education material that was provided to sites in the toolkit group. We will use methods published by Rex et al. to measure the cost of care [12]. The estimate will be based on the cost of the initial examination and assumptions about the first round of surveillance taking into consideration the adequacy of bowel preparation at the initial exam, clinical guidelines for surveillance, and Medicare charges for colonoscopy [10]. Patient outcomes include experience, attendance, and adherence. We will use an abbreviated version of the Ambulatory Care Experiences Survey (ACES), a validated survey that measures patients’ experience with a specific physician’s service including perceptions of health promotion support (2 items), service access (4 items), care coordination (2 items), and office staff interactions (2 items) [44]. Patient experience will be a composite score of these scales measured as a continuous variable. We will measure the context, facilitation, and evidence components of organizational readiness to change using an instrument administered to physician teams. Evidence will be measured using 8 items to assess the relative advantage and compatibility of the split-dose bowel preparation and low-literacy patient education interventions. Cronbach’s alpha for reliability of the evidence scale is 0.89 [45]. Sample items from this scale will ask physicians and staff their perceptions about whether using split-dose bowel preparation and low-literacy education with teach-back “is effective based on scientific evidence” and “takes into consideration the needs of patients.” These items will be measured on a Likert-type scale, with response options ranging from 1 = strongly disagree to 5 = strongly agree, and an “I don’t know/Not applicable” option. The context and facilitation components of organizational readiness to change will be assessed through a measure of quality of leader-member exchange (LMX) [32]. Staff will indicate the extent to which each item characterizes their exchange relationships with their supervisors. Sample items will include “How well does your supervisor understand your job problems and needs?” (1 = not at all, to 5 = fully) and “How would you characterize your working relationship with your supervisor?” (1 = extremely ineffective, to 5 = extremely effective).

### Google Analytics measures

To understand how the staff support website is used, we will leverage data tracked and analyzed via Google Analytics. As part of our pragmatic study, endoscopy staff members have flexibility in how they interact with the web-enhanced implementation toolkit. It is, therefore, particularly important to use rigorous yet unobtrusive measurements of staff website use. These data will serve as 1) an intervention check to assess whether or not staff members are using the website as intended and 2) an exploratory measure of website usability and usefulness that will inform future versions of the web-enhanced implementation toolkit. We will assess aggregated data on a number of use indicators, including sessions (i.e., period of time a user is actively engaged with the website), unique users (i.e., first-time users during the selected date range), pages per session, average session duration, average time per page, and website flow (i.e., illustration of the range of website interaction patterns). These data will also help inform qualitative assessment of the acceptability of the web-enhanced implementation toolkit. For instance, short session durations, low usage patterns for certain pages, and intended website flow behaviors will prompt targeted interview questions to expand on these initial quantitative findings.

### Analysis

If we find group-level effects, then we will dichotomize the readiness to change variables at mid-point (high, low) for the sample and analyze readiness to change at the physician-team level. We will examine the distribution of responses to the patient-experience survey for respondents overall and stratified by attendance. Our primary analysis for aim #2 will examine associations between each implementation and patient outcome and readiness to change using linear or logistic generalized estimating equations, depending on the distribution of the outcome, adjusted for patient and procedure characteristics. All analyses will be performed using SAS version 9.2. In addition, we will summarize data from aim #2 as six endoscopy setting case studies based on variability of clinical quality outcomes (aim #1) [46]. If we identify significant relationships between aspects of readiness to change and implementation, patient, or colonoscopy quality outcomes, then we will develop plans to analyze the patterns of these relations in a subsequent study that will be powered to address questions about pathways to readiness to change.

### Dissemination

We will disseminate study findings to scientific and practice stakeholders through manuscripts, presentations, and lay reports. We will also consult with our strategic partners to create physician-level reports that present colonoscopy quality outcomes compared to peers and quality goals. We will also adapt the implementation toolkit based on information gathered from follow-up surveys to improve its usefulness for ongoing support after the study is over and for use in a subsequent study that will examine wide dissemination of the toolkit to outpatient colonoscopy settings based on lessons learned.

### Ethical approval

Our study was found to be in compliance with the Helsinki Declaration and was approved by the Institutional Review Board (IRB) at Washington University’s Human Research Protection Office (IRB ID#: 201401089).

## Discussion

This study will uniquely test the effectiveness of two implementation strategies (physician education versus physician education plus an implementation toolkit for staff) in delivering three bundled EBPs (split-dosing of bowel preparation, low-literacy education, and teach-back) within a pragmatic study. Implementation challenges have already motivated several adaptations to the original plan, reflecting the nature of implementation in real-world healthcare settings. The pragmatic study responds to the evolving needs of its healthcare partners and allows for flexibility in intervention delivery, thereby informing clinical decision-making in real-world settings. Additionally, this comparative-effectiveness study accelerates the translation of research to practice by simultaneously informing stakeholders about the relative advantage of interventions and the strategic processes associated with successful implementation of the interventions. The current study will provide information about what works (effectiveness of the interventions), for whom it works (effectiveness for patients with Medicaid versus other insurance), in which contexts it works (aspects of the setting that influence implementation), and how it works best (physician education versus physician education plus staff training and support).

Additional potential challenges remain, including non-participation in the study. Therefore, we have taken steps to ensure maximum participation by engaging strategic partners in recruitment of physician teams and by planning multiple follow-ups with non-responders to the surveys. To minimize missing data, the research coordinator will check for missing responses as surveys are returned and contact respondents to clarify missing answers when key variables or more than 10 % of the data is missing. The validity of practice-based measures is further strengthened by triangulation of data sources [47–49]. For example, we measure fidelity and dose from physician team and patient perspectives, respectively, to learn about completion of intervention activities from multiple perspectives. This triangulation with multiple methods will add to the robustness and trustworthiness of the findings [49, 50], and when conflicts arise between findings from different sources, it will stimulate us to gather additional data to enhance understanding of the successful implementation processes.

## Consent

Written informed consent was obtained from participants for the publication of this report and any accompanying images.

## Additional file

Additional file 1:
**Additional information on the analytic model for the segmented regression analysis of interrupted time series data.**
