# Supplementary material for: Using segmented regression analysis of interrupted time series data to assess colonoscopy quality outcomes of a web-enhanced implementation toolkit to support evidence-based practices for bowel preparation: a study protocol
Source: Implement Sci. 2015 Jun 7;10:85. doi: 10.1186/s13012-015-0276-3 (PMC4465008; doi:10.1186/s13012-015-0276-3)
Supplement: Additional file 1: — Additional information on the analytic model for the segmented regression analysis of interrupted time series data. [file 13012_2015_276_MOESM1_ESM.docx]

**Additional file 1**

The model has a general form: Yt = β0 + β1*X1t + β2*X2t + β3*(X1t-C1)+ + β4*X3t + β5* (X1t – C2)+ + γ* Z (X1t ) + et where Yt is the proportion of adequate bowel preparation (or adenoma detection) at t, X1t is the time, a continuous variable indicating time at time t from the start of the observation period; X2t is an indicator taking value 0 before the comparative-effectiveness intervention and 1 after it; C1 is the time at which the comparative-effectiveness intervention study takes place. (X1t-C1)+ = Max((X1t-C1),0), taking the value of 0 if before the comparative-effectiveness intervention, and the number of 2-week intervals after it. X3t is an indicator taking value 0 before the replication study and 1 after it. C2 is the time at which the replication study takes place. (X1t-C2)+= Max((X1t-C2),0), taking the value of 0 before the replication study, and the number of 2-week intervals after it. Z(X1t ) is a vector of covariates (i.e. patient and procedure characteristics measured in the same aggregate level as outcomes). The et error term has some serial correlations. β0 estimates the level of outcome before the comparative-effectiveness intervention (baseline level), β1 estimates the change in outcome every 2-week interval before the comparative-effectiveness intervention (baseline trend). (β0 + β2) estimates the level of outcome after the comparative-effectiveness intervention but before the replication study, and (β1 + β3) estimates the change in outcome every 2-week interval after the comparative-effectiveness intervention but before the replication study. (β0 + β2 + β4) estimates the level of outcome after the replication study, and (β1 + β3 + β5) estimates the change in outcome every 2-week interval after the replication study. For group 2 which only has the comparative-effectiveness intervention, we do not have terms β4*X3t + β5* (X1t – C2)+ in the model.
